# Molecular Characterization of Previous Unclassified Dicistroviruses in Water Reservoir in São Paulo, Brazil

**DOI:** 10.3390/microorganisms14102403

**Published:** 2026-10-20

**Authors:** Mikaela Renata Funada Barbosa, Endrya do Socorro Foro Ramos, Fabiola Villanova, Renan Lourenço de Oliveira Silva, Wen Zhang, Noely Evangelista Ferreira, Tania Regina Tozetto-Mendoza, Maria Cássia Mendes-Correa, Ramendra Pati Pandey, Maria Inês Zanoli Sato, Elcio Leal, Antonio Charlys da Costa

**Affiliations:** 1 Department of Analysis, Environmental Company of the São Paulo State (CETESB), São Paulo 05459-900, SP, Brazil; mrbarbosa@sp.gov.br (M.R.F.B.); rlosilva@sp.gov.br (R.L.d.O.S.); misato@sp.gov.br (M.I.Z.S.); 2 General-Coordination of Public Health Laboratories, Health and Environment Surveillance Secretariat, Ministry of Health, Brasília 70723-040, DF, Brazil; endrya.ramos@gmail.com; 3 Viral Diversity Laboratory, Institute of Biological Sciences, Federal University of Pará, Belém 66077-830, PA, Brazil; fvillanova@gmail.com; 4 Department of Laboratory Medicine, School of Medicine, Jiangsu University, Zhenjiang 212013, China; z0216wen@yahoo.com; 5 Department of Infectious and Parasitic Diseases, Faculty of Medicine, University of São Paulo, São Paulo 05403-000, SP, Brazil; noely.evangelista@hc.fm.usp.br (N.E.F.); tozetto@usp.br (T.R.T.-M.); cassiamc@uol.com.br (M.C.M.-C.); 6 School of Biosciences and Bioengineering, D Y Patil International University, Akurdi, Pune 411044, Maharashtra, India; ramendra.pandey@ddn.upes.ac.in

**Keywords:** *Dicistroviridae*, freshwater, phylogenetics, virome, metagenomics

## Abstract

In this study, viral metagenomics was applied to identify viruses related to the *Dicistroviridae* family in freshwater samples collected from urban reservoirs of the state of São Paulo, Brazil. Through next-generation sequencing, five complete RNA genomes similar to *Dicistroviridae* were detected. These genomes range from 8.032 to 9.425 nucleotides in length, and each contains two open reading frames (ORFs) with conserved protein domains and internal ribosome entry sites (IRES) characteristic of *Dicistroviridae*. Phylogenetic and genetic identity analyses based on the amino acid sequences of the nonstructural (ORF1) and structural (ORF2) polyproteins suggest that these five genomes represent four putative novel species provisionally named Hydro virus 1 to Hydro virus 4. These findings enhance our understanding of the diversity and distribution of *Dicistroviridae* in aquatic environments.

## 1. Introduction

The *Dicistroviridae* family includes single-stranded RNA (ssRNA) viruses with linear genomes ranging in length from 8 to 10 kb and GC content of 34% to 45%, containing one or two non-overlapping reading frames (ORFs) separated by a conserved intergenic untranslated region (IGR-IRES). The ORF in the 5′ region encodes the non-structural proteins: RNA helicase (Hel), 3c-like protease (Pro), and RNA-dependent RNA polymerase (RdRP). Meanwhile, ORF2 in the 3′ region encodes the main structural proteins (VP1, VP2, VP3, and VP4). In addition, some members contain an additional ORF, called ORFx, within ORF2 [1].

Dicistroviruses are currently classified into three genera (*Aparavirus*, *Cripavirus*, and *Triatovirus*) based on the characteristics of the IGR-IRES and phylogenetic analysis. Regarding the demarcation of species, there are 16 recognized species, with new species sharing less than 90% identity between the capsid proteins [1,2].

Most members of the *Dicistroviridae* family infect arthropods, such as insects (bees, flies) and some crustaceans (shrimp). These viruses can be pathogenic to their hosts and cause significant economic impacts. For example, *Aparavirus tauraense* has been associated with problems in shrimp farming [3]. The viruses *Aparavirus israeli*, *Aparavirus kashmirense* (KBV), and *Aparavirus apisacutum* (ABPV) have implications for beekeeping, affecting honeybees [4,5].

Other species, such as *Cripavirus drosophilae* (DCV) [6], have been used as a model organism for genetic studies, while *Aparavirus vallesi* (SINV-1) [7] has potential as a biopesticide for controlling fire ant infestations.

Furthermore, *Triatovirus triatomae* (TrV), which infects triatomines (vectors of Chagas disease), may influence the transmission of *Trypanosoma cruzi* to humans [8,9]. However, the role of this virus in the epidemiology of Chagas disease has not yet been fully elucidated.

Furthermore, metagenomic studies have revealed new viral sequences related to this family (dicistro-like viruses) in different samples, including ants, cottony scale insects, bats, crustaceans [10,11,12,13,14,15,16,17], plants [18], and even environmental samples such as water [19,20].

Although many of these viruses still await official classification by the ICTV, the identification of new viral sequences is crucial for understanding the evolutionary history of the *Dicistroviridae* family. This advancement contributes to expanding knowledge about the group, its ecological interactions, and potential future biotechnological applications.

In this study, we describe the characterization of four new RNA viruses provisionally named Hydrovirus (HydVs) 1 through 4 (HydV1, HydV2, HydV3, and HydV4), which, based on genomic and phylogenetic evidence, likely represent new species within the *Dicistroviridae* family.

## 2. Materials and Methods

### 2.1. Collection of Water Samples

Surface freshwater samples were collected in September 2022 with support from the Basic Sanitation Company of the State of São Paulo (SABESP) from the Guarapiranga Reservoir (23°40′27″ S; 46°43′40″ W) and Taiaçupeba reservoirs (23°20′30″ S; 46°09′54″ W), located in the metropolitan region of São Paulo. Subsequently, the samples were transported on ice to the Virology Laboratory of the São Paulo State Environmental Company (CETESB) for processing within 24 h of collection, following the methodology previously described [21,22].

### 2.2. Virus Concentration Methods

Reservoir water samples (10 L) were concentrated by hollow-fiber ultrafiltration (HFUF) using a dead-end ultrafiltration (DEUF) system and polyethylene glycol (PEG) precipitation as previously described [21]. HdF100 S hemodialysis filters were pretreated with 0.01% sodium polyphosphate (NaPP), and retained viruses were eluted with a buffer containing Tween 80, NaPP, and Antifoam Y-30. Viral particles were further concentrated PEG precipitation by adding PEG 6000 and NaCl to the eluate, followed by overnight incubation at 4 °C and centrifugation. Pellets were resuspended in the residual volume, aliquoted, and stored at −80 °C until analysis.

Concentrated samples were pooled monthly by sampling site and treated with Vertrel XF (DuPont, Wilmington, DE, USA). After centrifugation, supernatants were filtered through 0.45-μm filters and treated with DNase to reduce nonviral nucleic acids. Total viral DNA/RNA was extracted either using the Extracta 16 automated platform (Loccus Biotecnologia, Cotia, São Paulo, Brazil) with magnetic beads (Promega, Madison, WI, USA) or the AllPrep PowerViral DNA/RNA Kit (Qiagen, Hilden, Germany), following the manufacturers’ instructions.

### 2.3. Nucleic Acid Extraction, Library Preparation, and Sequencing

Nucleic acid (DNA/RNA) was extracted from water samples using the Quick-DNA/RNA Pathogen MagBead kit (Zymo Research Corp., Irvine, CA, USA). For library construction, Nextera XT kits were used, following the manufacturer’s protocols. Purification and size selection steps were performed with the ProNex^®^ system (Promega, WI, USA) and Pippin Prep (Sage Science, Inc., Beverly, MA, USA), targeting fragments between 400 and 700 bp. Library quality was assessed with the Bioanalyzer 2100 (Agilent Technologies, Santa Clara, CA, USA), and quantification was performed to ensure equal representation in the pool. Sequencing was conducted on Illumina NovaSeq 6000 platforms (DNA, 500 paired-end cycles). Data were then processed using an in-house virus discovery pipeline at Jiangsu University School of Medicine, Zhenjiang, China [23]. Low-quality reads and primers were removed using Trim Galore v0.6.5 with specific parameters (–phred33 –length 50 –stringency 3 –paired). Paired-end reads were assembled with MEGAHIT v1.2.9 under default settings. Viral contigs were identified by comparison against a local non-redundant protein database (nr, May 2024). Contigs annotated as eukaryotic viruses were manually refined in Geneious Prime v2022.0.1 and used as references for read mapping to obtain complete or near-complete genomes.

Final assemblies were screened for vector contamination with VecScreen and clustered using MMseqs2 with stringent parameters (–e 0.001 –min-seq-id 0.95 –c 0.9). The resulting sequences were incorporated into a non-redundant viral dataset for downstream analyses.

### 2.4. Analysis of Assembled Contigs

Viral contigs were compared with full viral genomes in CheckV v1.5 to estimate completeness (0–100%) and viral genome quality: high (>90%), medium (50–90%), low (0–50%), or indeterminate [24].

Complete genomes were subjected to searches against the NCBI non-redundant (NR) protein database in BLAST version 2.17.0 [25], to select the best Best-Hit (with the highest percentage of identity and E-value < < 1 × 10^−5^ compared the sequences already deposited in Genbank).

### 2.5. Genome Annotation

Structural annotation of the genomes was performed using ORFfinder (https://www.ncbi.nlm.nih.gov/orffinder/, accessed on 2 September 2025) to predict open reading frames (ORFs). Protein domains and motifs of conserved proteins were predicted using the CDD Conserved Domain Database [26], Motif Finder (https://www.Genome.jp/tools/motif, 2 September 2025), Palmscan software 2.2 [27], and LucaProt [28]. For the prediction of IRES-IGR multiple sequence alignment (HydVs) with references, LocARNA [29,30] was used.

### 2.6. Recombination Screening

The presence of recombination events was evaluated using the RDP program (version 4.101), applying different available algorithms (RDP, GENECONV, BootScan, MaxChi, Chimaera, SiScan, and 3Seq). The alignment composed of HydV sequences and clades A to D, the Clirnapec virus 239 sequence (XII42478) was excluded from the recombination analysis due to its low quality. Only events detected by more than three methods with statistical significance (*p* < 0.05) were considered. No recombination events were identified.

### 2.7. Alignment and Phylogenetic Analysis

The nucleotide and amino acid sequences of this study along with cognate sequences and those of the *Dicistroviridae* family were aligned using the MUSCLE program [31] implemented in the UGENE program [32]. The IQ-Tree software 3.1.2 was used to infer maximum likelihood (ML) trees (Genome, ORF1 and ORF2) using the best-fit model (ModelFinder) [33]. Node support in the ML trees was assessed with 1000 ultrafast Bootstrap replicates. The generated trees were visualized by FigTree v 1.4. (https://github.com/rambaut/figtree/compare/v1.4.5pre...v1.5.0-beta1, accessed on 2 September 2025).

### 2.8. Evolutionary Distance

To assess the taxonomic assignment at the species level, the identity of the sequences identified in the study with sequences classified in the ICTV and those with greater homology were compared using the SDT program (v 1.2) [34]. Genetic distances and their standard errors were estimated in MEGA v12 (p-distance).

## 3. Results

Five genomes of positive-sense single-stranded RNA (ssRNA) viruses were recovered from water samples using a metagenomic approach followed by bioinformatic analysis. Details regarding library quality assessment including sequencing depth and coverage as are provided in the Supplementary Materials (Tables S1 and S2, Figures S1 and S2).

### 3.1. Organization of the HydVs Genome

These genomes were provisionally designated Hydro virus (HydVs) 1 to 4, including: HydV1 (SP_57630), HydV2 (SP_12745, SP_50593), HydV3 (SP_35161), and HydV4 (SP_4680). All identified genomes exhibited a linear organization, with sizes ranging from 8032 to 9425 nucleotides (nt) and GC content from 33.54% to 51.43%. Completeness analysis confirmed that each genome was fully assembled (Table S3). Sequence comparison using BLAST revealed the highest identity with unclassified viruses associated with the *Dicistroviridae* family (Tables S4 and S5), suggesting proximity to this group.

**Figure 1:**
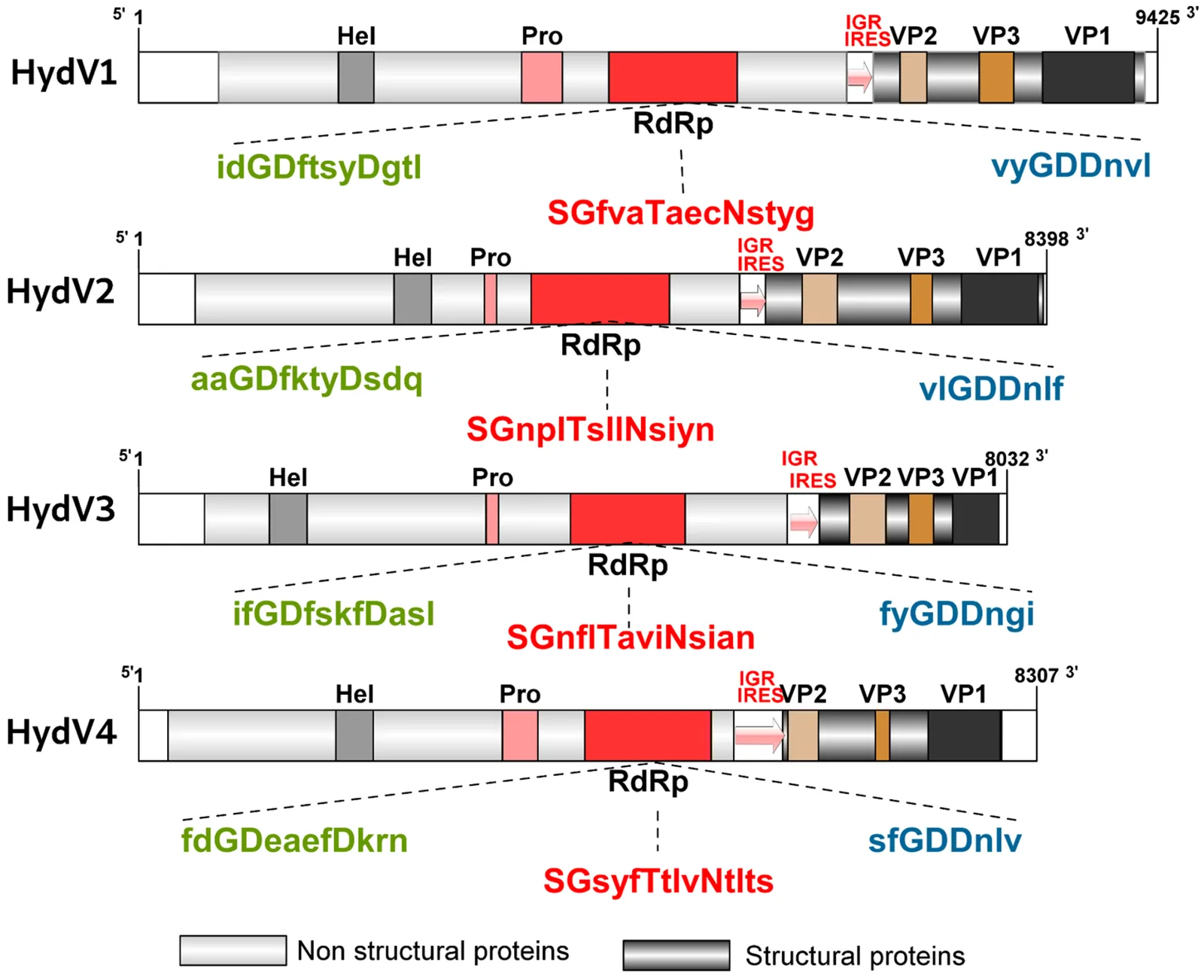
Genomic organization of HydVs. The genomes contain two non-overlapping ORFs separated by an IGR-IRES highlighted by a red arrow. ORF 1 encodes the non-structural proteins: RNA helicase (Hel; in gray), cysteine protease (Pro; in light red), and RNA-dependent RNA polymerase (RdRp; in red). ORF2 encodes the structural proteins VP1 (beige), VP2 (brown), and VP3 (black). The sequences of the RdRp motifs are highlighted in the image. Conserved amino acid residues are in uppercase and variable amino acids in lowercase. The sequence colors indicate motifs A (green), motif B (red), and motif C (blue).

Predictive structural annotation indicated that all genomes contained two non-overlapping ORFs separated by an IGR-IRES. ORF1 encodes non-structural proteins, including enzymes related to RNA replication and processing, whereas ORF2 encodes structural proteins presumably involved in viral capsid formation (Figure 1). This genomic architecture is consistent with patterns described for ssRNA+ viruses [35].

### 3.2. Domains and Motifs Detected in HydVs

Conserved domain analysis performed using the NCBI database and Motif Finder revealed that all sequences present in ORF1 share three characteristic domains: RNA helicase (PF00910), peptidase C3 (PF00548), and RNA-dependent RNA polymerase (RdRP; PF00680).

**Figure 2:**
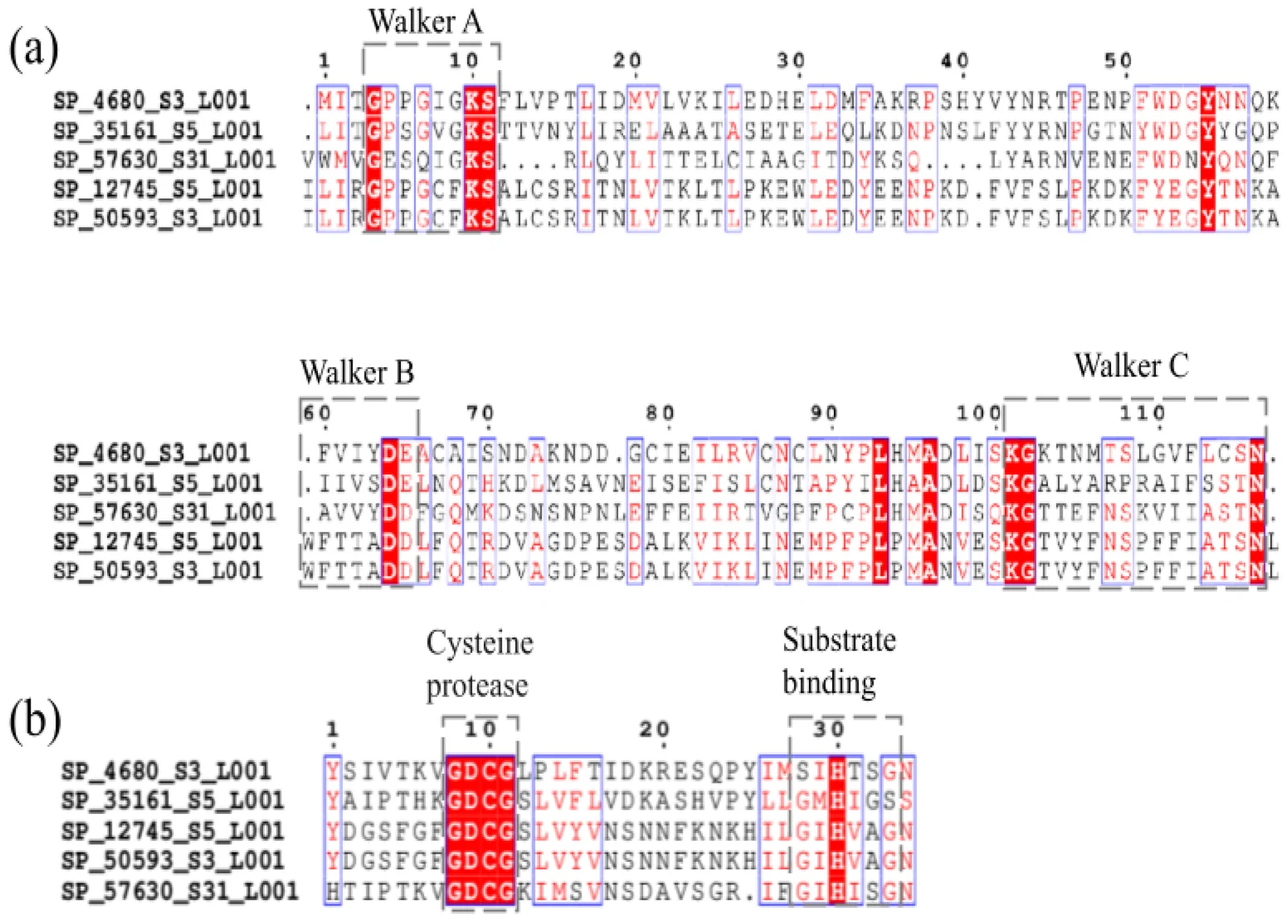
Multiple sequence alignments of HydVs domains. (**a**) Motifs provided in the Helicase domain. (**b**) Reasons predicted in the Protease domain. The colors of the residues shaded in red indicate completely conserved amino acids, residues in red are more than 80% conserved, and variable amino acids are in black. Conserved motifs are indicated by dashed boxes.

Within the helicase domain, the conserved motifs Walker A (GxxGxGKS/T), Walker B (x5DD), and Walker C (KgxxWxSxWWWx(S/T)(S/T)N) were detected, confirming the typical functionality of this group of enzymes (Figure 2a).

The protease domain exhibited the catalytic motifs GxCG and GxHxxG (Figure 2b), consistent with proteolytic activity essential for viral polyprotein processing.

Analysis of RdRP using LucaProt assigned the maximum probability (1.0) to its identification as a viral polymerase, validating the functional annotation [28]. The characteristic RdRP motifs A, B, and C were consistently detected, although amino acid variations were observed among the genomes, except sequences SP_12745 and SP_50593 (HydV2). This conservation suggests that both represent the same virus (HydV2).

Prediction of ORF2 indicated three structural proteins, VP2, VP3, and VP4, containing characteristic capsid domains, including RhV (PF00073), Dicistro_VP4 (PF11492), Calici_coat (PF00915), and CRPV_capsid (PF08762). This architecture reflects the typical genomic organization of members of the order *Picornavirales* [36,37]. Comparison of ORF2 sequences from HydV1 to HydV4 with officially recognized representatives of the *Dicistroviridae* family revealed sequence identities ranging from 21% to 30%, with alignment coverages from 30% to 99% (Table 1). These identity levels are substantially below the 90% threshold established by ICTV for species demarcation, corroborating the classification of HydV1 to HydV4 as a possible new species within the *Dicistroviridae* family.

#### HydVs IGR-IRES

To verify the taxonomic relationship of HydVs with established genera (*Aparavirus*, *Cripavirus*, and *Triatovirus*), the sequences of the probable IGR-IRES were analyzed. Members of the *Dicistroviridae* family have IGR-IRES sequencens of approximately 200 nucleotides in length. This region is responsible for directing the translation of ORF2, based on the recognition of a non-AUG start codon [2,38].

**Figure 3:**
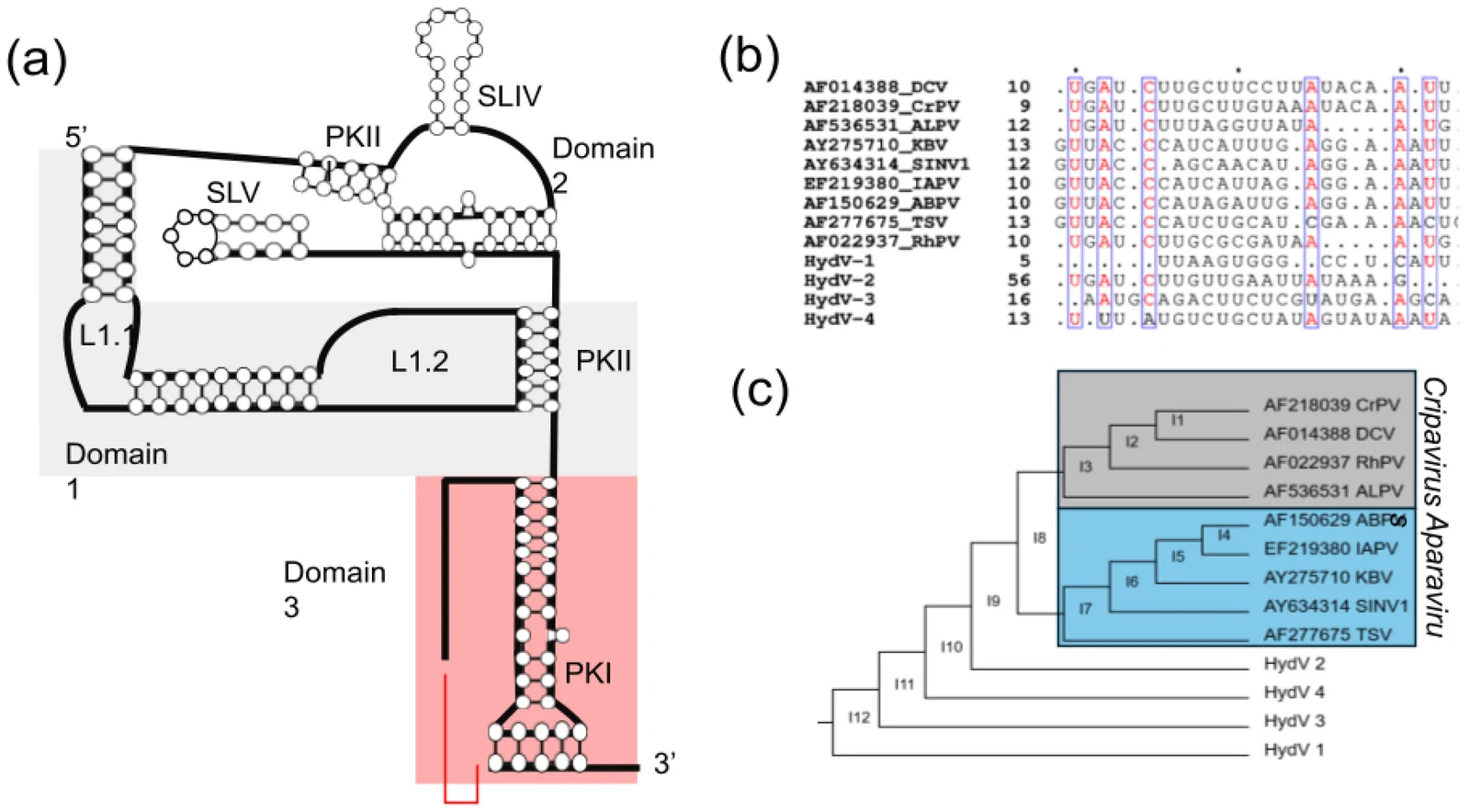
Multiple sequence alignments of HydVs domains. (**a**) Diagram of the IRES-IGR elements, the PKI, PKII, and PKIII pseudoknots; the SLIV and SLV stem loops, and the L1.1 and L1.2 loops are indicated. (**b**) Multiple alignment of IGR-IRES sequences of HydVs, compared with virus species of the *Dicistroviridae* family ALPV: *Cripavirus mortiferum*; CrPV: *Cripavirus grylli*; DCV: *Cripavirus drosophilae*; RhPV: *Cripavirus ropadi*; ABPV: *Aparavirus_apisacutum*; KBV: *Aparavirus kashmirense*; IAPV: *Aparavirus israeli*; TSV: *Aparavirus tauraense* and SINV1: *Aparavirus vallesi*. Residues in red indicate more than 80% conservation, variable amino acids are in black. (**c**) Guide tree of the alignment performed in the LocARNA software version 2.0.0 showing the hierarchical grouping of the sequences, in which the HydVs are not within the *Cripavirus* and *Aparavirus* clades. These clades are indicated in the tree by gray and blue boxes.

The IGR-IRES is formed by a conserved structure composed of three domains. Domain 1 contains the pseudoknot (PK) III and two trunk loops (SLIV and SLV) exposed, containing conserved motifs that interact with the 40S subunit. Domain 2 is composed of PK II and loop 1.1 (L1.1), which is responsible for interaction with the L1 stem of the large 60S ribosomal subunit. While domain 3PK I is responsible for the correct positioning of viral RNA to the P site of the small ribosomal subunit and the variable loop region (VLR) [2] (Figure 3a).

Analysis of the HydVs IGR-IRES showed regions with similarity to other *Dicistroviridae* (Figure S3). Two sequences (SP_12745 and SP_50593, HydV2) were predicted to contain the bulge sequence (UGAUCU), similar to the *Cripavirus* IRES (Figure 3b), the alignment showed differences. HydV2 did not group with representatives of the *Cripavirus* genus (Figure 3c). The sequences UGGUUACCCAU and UAAGGCUU of *Aparavirus* and conserved ternary motif (DDF) were not detected in any of the sequences.

From a structural perspective, HydV IRES elements are shorter (120–253 nt), with fewer hairpins (3–5) and less negative free energy values (−23 to −52 kcal/mol), suggesting lower stability. In contrast, reference viruses of the genus *Cripavirus*, represented by *Cripavirus grylli* (CrPV), *Cripavirus drosophilae* (DCV), *Cripavirus ropadi* (RhPV), and *Cripavirus mortiferum* (ALPV), exhibit IRES of intermediate length (170–186 nt), with 3–4 hairpins and multiple internal loops. Their free energy values range from −34 to −43 kcal/mol, indicating moderate stability.

In comparison, the IRES of *Aparaviruses*, including *Aparavirus apisacutum* (ABPV), *Aparavirus israeli* (IAPV), *Aparavirus kashmirense* (KBV), *Aparavirus vallesi* (SINV1), and *Aparavirus tauraense* (TSV), possess more complex structures, with 181–187 nt, five hairpins, and numerous internal loops. Their free energy values are more negative (−52 to −69 kcal/mol), indicating greater structural stability.

These quantitative and qualitative differences suggest that HydV IRES elements do not fall within the known intrageneric variability of these genera, potentially representing a distinct group within the *Dicistroviridae* family. This finding expands the structural and functional diversity of IRES elements and highlights the need for further research.

### 3.3. Phylogenetic Inference Based on ORF1 and ORF2

Phylogenetic analyses conducted with sequences from the non-structural region (ORF1) and the structural region (ORF2) of viruses identified in aquatic samples, together with representatives of the *Dicistroviridae* family and unclassified viruses, validated the differences observed in the IGR-IRES. In addition, recombination screening performed with the RDP program did not reveal any signs of recombination among the genomes analyzed (sequences HydVs and *Dicistroviridae* reference sequences), further supporting the robustness of the phylogenetic interpretation.

**Figure 4:**
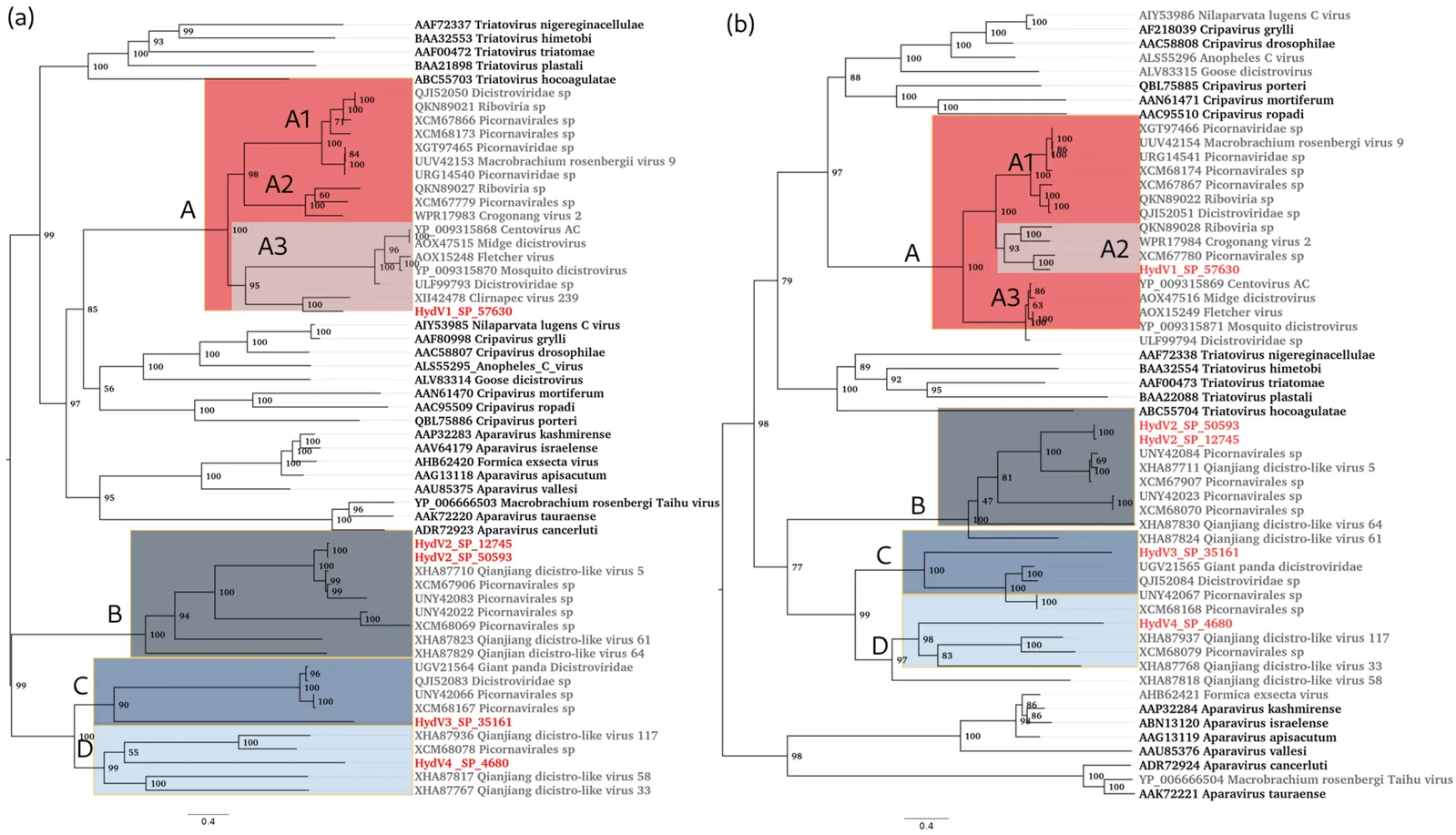
Phylogenetic analysis of HydVs based on amino acid sequences of viruses classified within the Dicistroviridae family. Phylogenetic trees were constructed using the maximum likelihood approach implemented in IQ-TREE. (**a**) ORF1 tree, inferred with the VT + F + R8 model, based on 57 sequences with an alignment length of 1103 amino acids. (**b**) ORF2 tree, inferred with the LG + F + R5 model, based on 55 sequences (excluding the partial XII42478 Clirnapec virus 239) with an alignment length of 635 amino acids. HydVs are indicated in red font, previously classified viruses in black, and unclassified viruses in gray. Clades containing the novel sequences are highlighted with the colored squares that are indicated by capital letters. Numbers at each node represent bootstrap support values (expressed as percentages from 1000 replicates), providing a measure of the robustness of each branching. Scale bars indicate the number of substitutions per site, allowing direct interpretation of branch lengths.

The HydVs sequences are consistently clustered within *Dicistroviridae*, forming branches supported by bootstrap values exceeding 70, yet positioned in clades distinct from the currently recognized genera (Figure 4a,b).

The phylogenetic reconstruction positioned HydV1 (SP_57630) within Clade A, subdivided into three distinct subclades. The placement of HydV1 varied between genomic regions: in ORF1, it clustered within subclade A3, showing the highest homology with the partial sequence of *Clirnapec virus 239* (XII42478), identified in *Ortmanniana pectorosa* in the United States. In contrast, in ORF2, it was placed in subclade A2, more closely related to an unclassified virus described in a 2017 metagenomic survey of water samples from the Teltow Canal, Germany [19,39]. The absence of recombination in the sequences (HtdV1, PP872618, MT138411, OR270207, NC_031687, KX782310, NC_031688, and MZ679073) suggests that the observed differences reflect distinct selective pressures acting on structural capsid proteins, which exhibit greater evolutionary variability compared to the replicative region.

The sequences HydV2 (SP_12745 and SP_50593) were closely related, clustering within Clade B alongside other sequences derived from aquatic samples collected in Germany [19,20] and from invertebrates (*Procambarus clarkii*) sampled in China in 2021.

HydV3 (SP_35161) was assigned to Clade C, forming an independent branch closely related to the *Giant panda dicistrovirus* described in intestinal metagenomics of *Ailuropoda melanoleuca* [40], and also included unclassified sequences predominantly derived from aquatic environments.

Finally, HydV4 (SP_4680) clustered within Clade D, showing the highest homology with *Qianjiang dicistro-like virus 117* identified in *Procambarus clarkii* in China, together with other aquatic sequences [19]. Genome-based phylogenies corroborated the topology observed for ORF2 (Figure S4).

### 3.4. Comparisons of Genetic Identity

Pairwise comparisons of amino acid sequences from RdRp (ORF1) and capsid (ORF2) revealed low identity (<40%) relative to members of the genera *Aparavirus*, *Cripavirus*, and *Triatovirus* across both regions (Figures S5 and S6). Notably, sequences HydV1 to HydV4 shared 78.32%, 60.92%, 38.78%, and 28.06% identity in ORF2 with their closest relatives (XCM67780, XCM67907, UGV21565, and XHA87936, respectively). These findings strongly support the classification of these isolates as novel viral species.

### 3.5. Genetic Distance Analyses

To assess the extent of evolutionary divergence, we calculated pairwise genetic distance matrices among clades A–D, in which HydV sequences cluster, based on amino acid substitutions in both the RNA-dependent RNA polymerase (ORF1) and the capsid protein (ORF2).

Analysis of ORF1 revealed mean pairwise distances of 0.49 ± 0.001 for clade A1, 0.23 ± 0.01 for A2, and 0.15 ± 0.01 for A3. In the more divergent clades, we observed 0.52 ± 0.01 in B, 0.38 ± 0.01 in C, and 0.69 ± 0.01 in D. In contrast, ORF2 analysis showed lower values in closely related clades: 0.35 ± 0.01 in A1, 0.16 ± 0.01 in A2, and only 0.06 ± 0.01 in A3. The more diverse clades exhibited 0.49 ± 0.01 in B, 0.06 ± 0.04 in C, and 0.62 ± 0.01 in D.

Comparisons between HydV clades and established *Dicistroviridae* genera reinforced this trend. In the RdRp (ORF1), mean distances were 0.78–0.80 for A1, 0.81–0.82 for B and C, and 0.79–0.80 for D. In ORF2, values ranged from 0.69–0.78 for A1, 0.77–0.79 for B, 0.77–0.80 for C, and 0.76–0.80 for D.

These levels of divergence, greater than those observed in recognized genera, may suggest that HydVs might not be accommodated within currently defined taxonomic classifications. However, this is a preliminary hypothesis that requires further validation according to ICTV criteria, and analyses point to the need for broader taxonomic criteria capable of capturing the diversity of dicistrovirus-like sequences that remain unclassified by ICTV.

## 4. Discussion

The investigation of viruses identified in freshwater samples, collected at the Guarapiranga and Taiaçupeba reservoirs in the state of São Paulo, revealed distinct genetic and phylogenetic characteristics. These viruses presented genomic organization consistent with the order *Picornavirales* containing the typical replication block (Hel, Pro and RdRp) and the presence of IGR-IRES, similar to other members of the *Dicistroviridae* family [1].

Sequence comparison showed low genetic identity (<90%) compared to species previously classified by ICTV and those with higher homology in BLAST. The ICTV currently uses 90% amino acid sequence identity in the cap region as a demarcation criterion for species within the *Dicistroviridae* family [1]. Based on these criteria, the sequences represent four putative novel species in the *Dicistroviridae* family, provisionally and informally named Hydro virus 1 to Hydro virus 4 (HydV1 a HydV4). It is important to highlight that BLASTn results showing high identity, but extremely low coverage (e.g., 93–98% identity with 1% coverage, Table S5) reflect only conserved fragments and do not indicate significant genomic proximity. This clarification avoids misinterpretation and supports the classification of HydVs as distinct species.

Phylogenetic analyses reinforce this interpretation by showing that HydVs cluster separately from the three established genera within *Dicistroviridae*, supporting the differences observed in the IGR-IRES sequences (Figure S3). These data suggest that HydVs may represent lineages distinct from the current taxonomic classifications. However, this remains a preliminary hypothesis, as confirmation of their taxonomic placement depends on official evaluation by the ICTV.

Other investigations have also described dicistrovirus-like viruses not yet classified by the ICTV, positioned in clades distinct from the recognized genera [16,41]. This highlights the challenge of classifying new members of *Dicistroviridae* based on current taxonomic criteria, which, although useful, may not be sufficient to accommodate the diversity revealed by metagenomic studies, reinforcing the need for broader criteria [41].

From the perspective of environmental surveillance, the detection of HydVs in freshwater reservoirs broadens this discussion by highlighting the strategic role of these environments as key points for a better understanding of viral diversity. This observation is consistent with metagenomic studies in water samples, which have emphasized the existence of a viral diversity much greater than currently recognized and still largely unexplored, particularly with regard to the ecological role and infectivity of these viruses aspects that have not yet been elucidated [41,42,43].

To date, dicistroviruses have been reported in association with different invertebrates, including insects, arthropods, crustaceans, and plants [19,20,41,43]. In this study, we hypothesize that HydVs may have the capacity to infect crustaceans, since the closest sequences identified in comparative analyses were reported in this type of organism [19,20]. Nevertheless, it is still unclear whether crustaceans are truly direct hosts of these viruses, as they may acquire viral RNA fragments indirectly, for example, through predation of other organisms or via parasites.

In general the evolution of RNA viruses is strongly influenced by horizontal transfer between different hosts [44]. This means that species sharing the same environment can carry similar viruses, even without being closely related. In the case of dicistroviruses, phylogenetic analyses previous suggest that their evolutionary trajectory reflects both the ecology and evolutionary history of their hosts, indicating a transition from aquatic to terrestrial environments and, possibly, from insects to plants [43]. Despite these inferences, the natural hosts of dicistrovirus-like viruses such as HydVs remain unconfirmed. To establish this, additional experiments are required, including inoculation assays in potential hosts, monitoring of viral replication in specific tissues, and analyses of host responses.

The broad diversity of dicistrovirus-like viruses already described in different environments, including water samples, reinforces the importance of exploring viral diversity [15,19,20,42,43,44,45,46,47] to identify and characterize new viruses, refine taxonomic criteria, and provide insights into the origins and evolution of *Dicistroviridae*.

In conclusion, this study describes a new viral species with genomic organization and phylogenetic characteristics similar to those of the family *Dicistroviridae*, highlighting the significant genetic variability of this group. The results also emphasize the importance of viral surveillance in aquatic environments, both to detect new or already known viruses and to better understand the role of viruses in biodiversity and the ecology of these ecosystems.

## Figures and Tables

**Table 1:** Amino acid identity of HydVs ORF2 compared with representative *Dicistroviridae* species.

Accession Number	Species	HydV1		HydV2		HydV3		HydV4	
		Identity (%)	Coverage (%)	Identity (%)	Coverage (%)	Identity (%)	Coverage (%)	Identity (%)	Coverage (%)
*AAG13119*	*Aparavirus apisacutu*	21.75	78	25.48	78	25.09	30	23.07	71
*ADR72924*	*Aparavirus cancerluti*	24.15	60	23.72	53	21.38	40	25.39	57
*ABN13120*	*Aparavirus israelense*	22.61	82	25.23	84	26.15	24	22.46	76
*AAP32284*	*Aparavirus kashmirense*	25.71	64	27.10	59	22.40	54	24.72	75
*AAK72221*	*Aparavirus tauraense*	24.26	57	24.88	56	24.54	41	24.13	49
*AAU85376*	*Aparavirus vallesi*	26.32	54	24.53	68	21.58	31	24.93	48
*AAC58808*	*Cripavirus drosophilae*	28.99	93	26.46	62	22.85	51	24.88	72
*AF218039*	*Cripavirus grylli*	30.53	93	24.44	95	21.55	56	25.89	63
*AAN61471*	*Cripavirus mortiferum*	29.56	93	29.71	79	24.36	72	24.60	74
*QBL75885*	*Cripavirus porteri*	28.06	99	28.24	82	24.23	95	30.97	61
*AAC95510*	*Cripavirus ropadi*	30.46	86	25.84	75	25.07	84	27.02	68
*BAA32554*	*Triatovirus himetobi*	26.96	77	24.14	82	24.47	47	24.33	72
*ABC55704*	*Triatovirus hocoagulatae*	24.77	74	22.46	81	23.32	59	23.64	69
*AAF72338*	*Triatovirus nigereginacellulae*	26.62	67	25.37	76	24.04	63	26.51	72
*BAA22088*	*Triatovirus plastali*	26.75	79	24.36	83	25.23	64	25.82	72

## Data Availability

Clean read data has been deposited in the database (PRJNA1328376). Viral genome sequences have been deposited in the NCBI GenBank database (PX241861-PX241865).

## Supplementary Materials

The following supporting information can be downloaded at: https://www.mdpi.com/article/10.3390/microorganisms14102403/s1, Table S1. Sequencing and metagenomic assembly data by library. Table S2. Assembly support metrics for viral contigs. Table S3. Data HydV detected in water samples in the state of São Paulo. Table S4. Summary of Identified Viral Contigs and Genomic Quality. Table S5. Nucleotide comparison of HydV sequences in BLASTn and BLASTx. Table S6. Amino acid comparison of HydV sequences in BLASTp. Table S7. Individual sequence metrics of IGR-IRES across Dicistroviridae reference viruses and HydV. Figure S1. Reads assigned to the family *Dicistroviridae* relative to other viral taxa in the three metagenomic libraries. Figure S2. The fifteen most abundant viral families in the SRR35404535, SRR27256804, and SRR27256806 libraries classified with Kraken 2. Figure S3. Multiple sequence alignment of the IRES-IGR region of HydV. Figure S4. Maximum likelihood trees of the complete genomes. Figure S5. Pairwise identity matrix of HydV sequences (ORF2). Figure S6. Pairwise identity matrix of HydV sequences (ORF1).
