# Supplementary material for: Molecular Characterization of Previous Unclassified Dicistroviruses in Water Reservoir in São Paulo, Brazil
Source: Microorganisms. 2026 Oct 20;14(10):2403. doi: 10.3390/microorganisms14102403 (PMC13707985; doi:10.3390/microorganisms14102403)
Supplement: Supplementary file 1 [file microorganisms-14-02403-s001.zip › microorganisms-4583505-supplementary.pdf]

**Supplementary Material**

# Molecular characterization of previous unclassified dicistroviruses in water reservoir in São Paulo, Brazil

**Mikaela Renata Funada Barbosa [1,†], Endrya do Socorro Foro Ramos [2,†], Fabiola Villanova [3], Renan Lourenço de Oliveira Silva [1], Wen Zhang [4], Noely Evangelista Ferreira [5], Tania Regina Tozetto-Mendoza [5], Maria Cássia Mendes-Correa [5], Ramendra Pati Pandey [6], Maria Inês Zanoli Sato [1], Elcio Leal [3,*,‡] and Antonio Charlys da Costa [5,*,‡]**

[1] Department of Analysis, Environmental Company of the São Paulo State (CETESB), São Paulo 05459-900, SP, Brazil; mrbarbosa@sp.gov.br (M.R.F.B.); rlosilva@sp.gov.br (R.L.d.O.S.); misato@sp.gov.br (M.I.Z.S.)

[2] General-Coordination of Public Health Laboratories, Health and Environment Surveillance Secretariat, Ministry of Health, Brasília 70723-040, DF, Brazil; endrya.ramos@gmail.com

[3] Viral Diversity Laboratory, Institute of Biological Sciences, Federal University of Pará, Belém 66077-830, PA, Brazil; fvillanova@gmail.com

[4] Department of Laboratory Medicine, School of Medicine, Jiangsu University, Zhenjiang 212013, China; z0216wen@yahoo.com

[5] Department of Infectious and Parasitic Diseases, Faculty of Medicine, University of São Paulo, São Paulo 05403-000, SP, Brazil; noely.evangelista@hc.fm.usp.br (N.E.F.); tozetto@usp.br (T.R.T.-M.); cassiamc@uol.com.br (M.C.M.-C.)

[6] School of Biosciences and Bioengineering, D Y Patil International University, Akurdi, Pune 411044, Maharashtra, India; ramendra.pandey@ddn.upes.ac.in

* Correspondence: elcioleal@ufpa.br (E.L.); charlysbr@yahoo.com.br (A.C.d.C.)

† These authors contributed equally to this work.

‡ These authors jointly supervised this work.

## TABLES:

**Table S1.** Sequencing and metagenomic assembly data by library.

| Library | Total reads (PAIRED) | Number of filtered reads | Number of contigs generated | Average Size (pb) |
|---|---|---|---|---|
| SRR35404535 (S31) | 1.931.959 | 1.771.203 | 79.103 | 575,7 |
| SRR27256804 (S5) | 6.946.362 | 6.908.410 | 132.092 | 351,3 |
| SRR27256806 (S3) | 6.481.362 | 6.445.320 | 148.955 | 516,1 |

**Table S2.** Assembly support metrics for viral contigs.

| Contig | Total Reads | Mapped Reads | Genome Coverage ≥1× | Mean Depth (×) | Mean Base Quality | Mean Mapping Quality |
|---|---|---|---|---|---|---|
| SP_57630 (HydV1) | 1.931.959 | 9036 | 100% | 145× | 35.2 | 35.7 |
| SP_12745 (HydV2) | 6.946.362 | 2.016 | 100% | 57,5x | 33.3 | 1,12 |
| SP_50593 (HydV2) | 6.481.362 | 2.089 | 100% | 58,1× | 33.3 | 0.87 |
| SP_35161 (HydV3) | 6.946.362 | 2.103 | 100% | 38× | 33.4 | 34.5 |
| SP_4680 (HydV4) | 6.481.362 | 3.095 | 100% | 55× | 33.3 | 34,8 |

**Table S3.** Data HydV detected in water samples in the state of São Paulo**.**

| Contig_ID (Virus name ) | Sampling Point | Latitude / Longitude | Collection date |
|---|---|---|---|
| SP_57630 (HydV1) | PEBA Taiaçupeba reservoirs | (23°20'30" S; 46°09'54" W) | 2022 |
| SP_12745 (HydV2) | Guarapiranga Reservoir | (23°40’27’’ S; 46°43’40’’ W) | 2022 |
| SP_50593 (HydV2) | Guarapiranga Reservoir | (23°40’27’’ S; 46°43’40’’ W) | 2022 |
| SP_35161 (HydV3) | Guarapiranga Reservoir | (23°40’27’’ S; 46°43’40’’ W) | 2022 |
| SP_4680 (HydV4) | Guarapiranga Reservoir | (23°40’27’’ S; 46°43’40’’ W) | 2022 |

**Table S4.** Summary of Identified Viral Contigs and Genomic Quality

| Contig_ID (Name virus) | Library code | Length (bp) | GC Content | Genome Completeness | Checkv_quality |
|---|---|---|---|---|---|
| SP_57630 (HydV1) | S31_L001 | 9425 nt | 51.43% | CG – 100% | High-quality |
| SP_12745 (HydV2) | S5_L001 | 8360 nt | 35.81% | CG - 92.79% | High-quality |
| SP_50593 (HydV2) | S3_L001 | 8398 nt | 35.85% | CG - 93.21% | High-quality |
| SP_35161 (HydV3) | S5_L001 | 8032 nt | 46.40% | CG - 90.00% | High-quality |

| | | | | | |
|---|---|---|---|---|---|
| SP_4680 (HydV4) | S3_L001 | 8307 nt | 33.54% | CG - 95.15% | High-quality |

**Table S5**. Nucleotide comparison of HydV sequences in BLASTn and BLASTx.

| Contig_ID | BLASTn | | | | BLASTx | | | |
|---|---|---|---|---|---|---|---|---|
| | **Best-hit** | **Identity** | **Coverage** | **E-values** | **Best-hit** | **Identity** | **Coverage** | **E-values** |
| SP_57630 (HydV1) | OR270498 | 77.94% | 19% | 0.0 | XII42478 | 64.46% | 56% | 0.0 |
| SP_12745 (HydV2) | PQ054865 | 77.55% | 67% | 0.0 | XCM67906 | 82.11% | 59% | 0.0 |
| SP_50593 (HydV2) | PQ054865 | 77.55% | 68% | 0.0 | XCM67906 | 82.11% | 60% | 0.0 |
| SP_4680 (HydV3) | LC662988 | 93.62% | 1% | 9e-06 | XHA87767 | 28.72% | 47% | 3e-140 |
| SP_35161 (HydV4) | LC662870 | 98.57% | 1% | 2e-22 | URG14820 | 33.29% | 31% | 4e-137 |

**Table S6.** Amino acid comparison of HydV sequences in BLASTp.

| Contig_ID | BLASTp (ORF1) | | | | BLASTp (ORF2) | | | |
|---|---|---|---|---|---|---|---|---|
| | **Best-hit** | **Identity** | **Coverage** | **E-values** | **Best-hit** | **Identity** | **Coverage** | **E-values** |
| SP_57630 (HydV1) | XII42478 | 64.46% | 91% | 0.0 | XCM67780 | 77.82% | 100% | 0.0 |
| SP_12745 (HydV2) | XCM67906 | 82.11% | 100% | 0.0 | XCM67907 | 60.21% | 98% | 0.0 |
| SP_50593 (HydV2) | XCM67906 | 82.11% | 100% | 0.0 | XCM67907 | 60.21% | 98% | 0.0 |

| | | | | | | | | |
|---|---|---|---|---|---|---|---|---|
| SP_35161 (HydV3) | URG14820 | 33.22% | 50% | 2e-141 | UGV21565 | 35.26% | 99% | 8e-115 |
| SP_4680 (HydV4) | XHA87767 | 28.92% | 72% | 7e-148 | XHA87937 | 39.87% | 91% | 5e-129 |

**Table S7.** Individual sequence metrics of IGR-IRES across *Dicistroviridae* reference viruses and HydV.

| **Virus/Sequence** | **Group** | **Length (nt)** | **Hairpins** | **ΔG (kcal/mol)** | **Conserved Motifs** |
|---|---|---|---|---|---|
| CrPV | Cripavirus | 186 | 4 | –39 | UGAUCU |
| DCV | Cripavirus | 170 | 3 | –34 | UGAUCU |
| RhPV | Cripavirus | 170 | 4 | –43 | UGAUCU |
| ALPV | Cripavirus | 172 | 3 | –35 | UGAUCU |
| ABPV | Aparavirus | 187 | 5 | –58 | UGGUUACCCAU, UAAGGCUU, DDF |
| IAPV | Aparavirus | 187 | 5 | –69 | UGGUUACCCAU, UAAGGCUU, DDF |
| KBV | Aparavirus | 187 | 5 | –61 | UGGUUACCCAU, UAAGGCUU, DDF |
| SINV1 | Aparavirus | 185 | 5 | –52 | UGGUUACCCAU, UAAGGCUU, DDF |
| TSV | Aparavirus | 181 | 5 | –56 | UGGUUACCCAU, UAAGGCUU, DDF |
| HydV1 | HydV | 120 | 3 | –23 | None detected |
| HydV2 (SP_12745) | HydV | 253 | 5 | –52 | Partial UGAUCU (not clustered) |
| HydV3 (SP_50593) | HydV | 198 | 4 | –41 | None detected |
| HydV4 | HydV | 175 | 4 | –36 | None detected |

# FIGURES

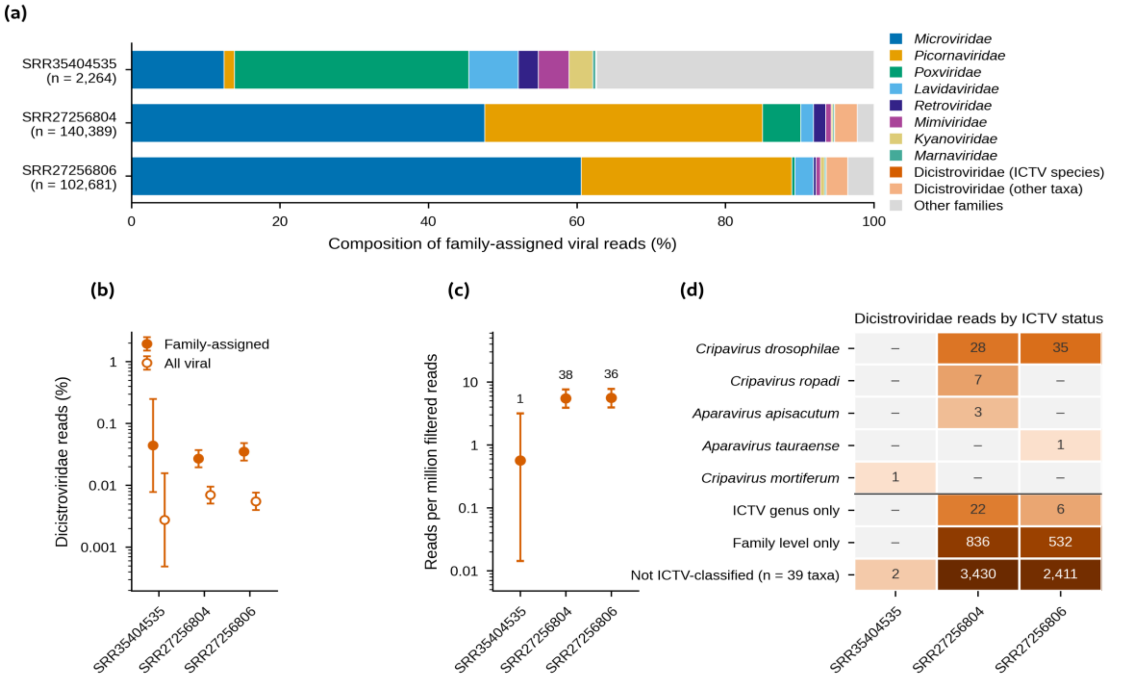


**Figure S1.** Reads assigned to the family *Dicistroviridae* relative to other viral taxa in the three metagenomic libraries. a) Family-level composition of viral reads from libraries SRR35404535 (S31), SRR27256804 (S5), and SRR27256806 (S3) classified with Kraken2 (Viral database, 2025) and summarized with Kraken Tools. n is the total number of viral reads with an assigned family. Counts correspond to reads assigned directly to each taxon at the family level. The eight most abundant families across the libraries are displayed individually, along with Dicistroviridae, and the remaining families are grouped as other families. b) Proportion of Dicistroviridae reads among viral reads with family-level assignment (filled circles) and among all viral reads (open circles); error bars, 95% confidence intervals. c) Dicistroviridae reads per million total reads; n, number of Dicistroviridae reads; error bars, exact 95% Poisson confidence intervals. d) Number of reads assigned to each Dicistroviridae taxon per library; dashes indicate the absence of assigned reads.

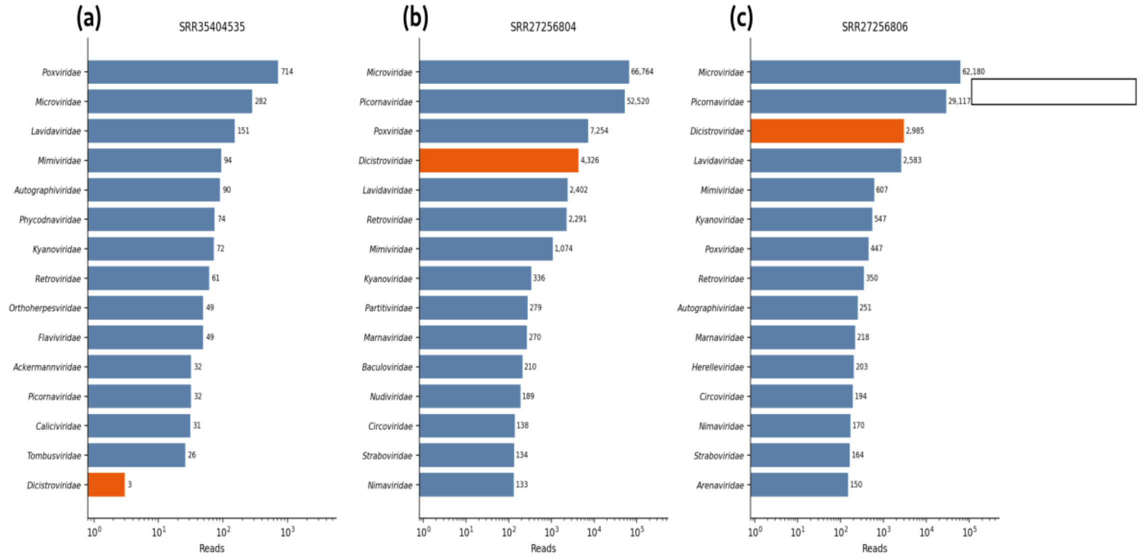


**Figure S2**. The fifteen most abundant viral families in the SRR35404535, SRR27256804, and SRR27256806 libraries classified with Kraken 2. The family *Dicistroviridae* (orange) is displayed and replaces the 15th family when the latter occupies a lower position in the ranking. Bars indicate read counts on a logarithmic scale; values represent the number of reads assigned to each family.

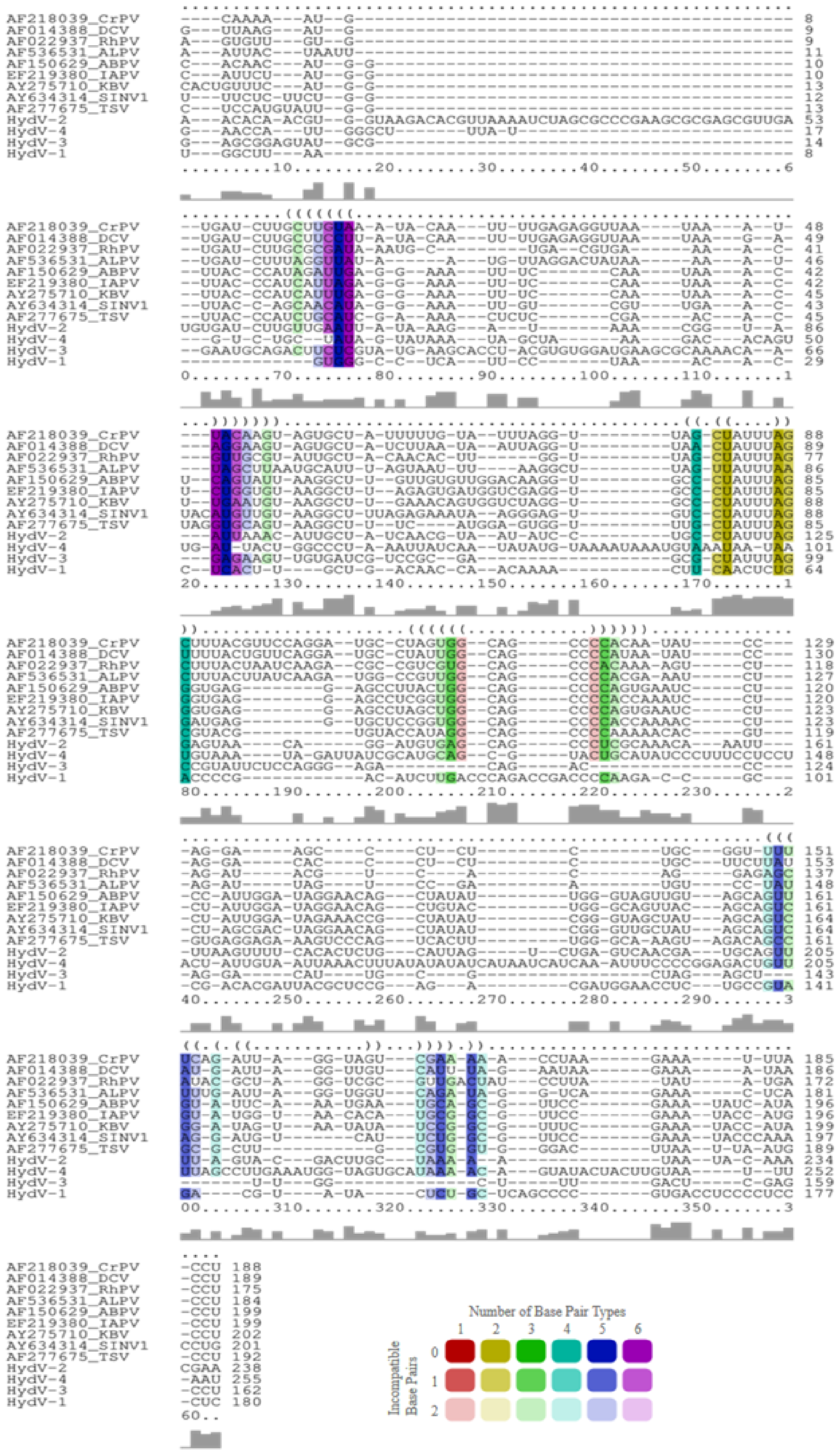

**Figure S3**. Multiple sequence alignment of the IRES-IGR region of HydV. Sequences with members of *Dicistroviridae* using the LocARNA software. Colors indicate base pair conservation between sequences (CG, GC, AU, UA, GU or UG).

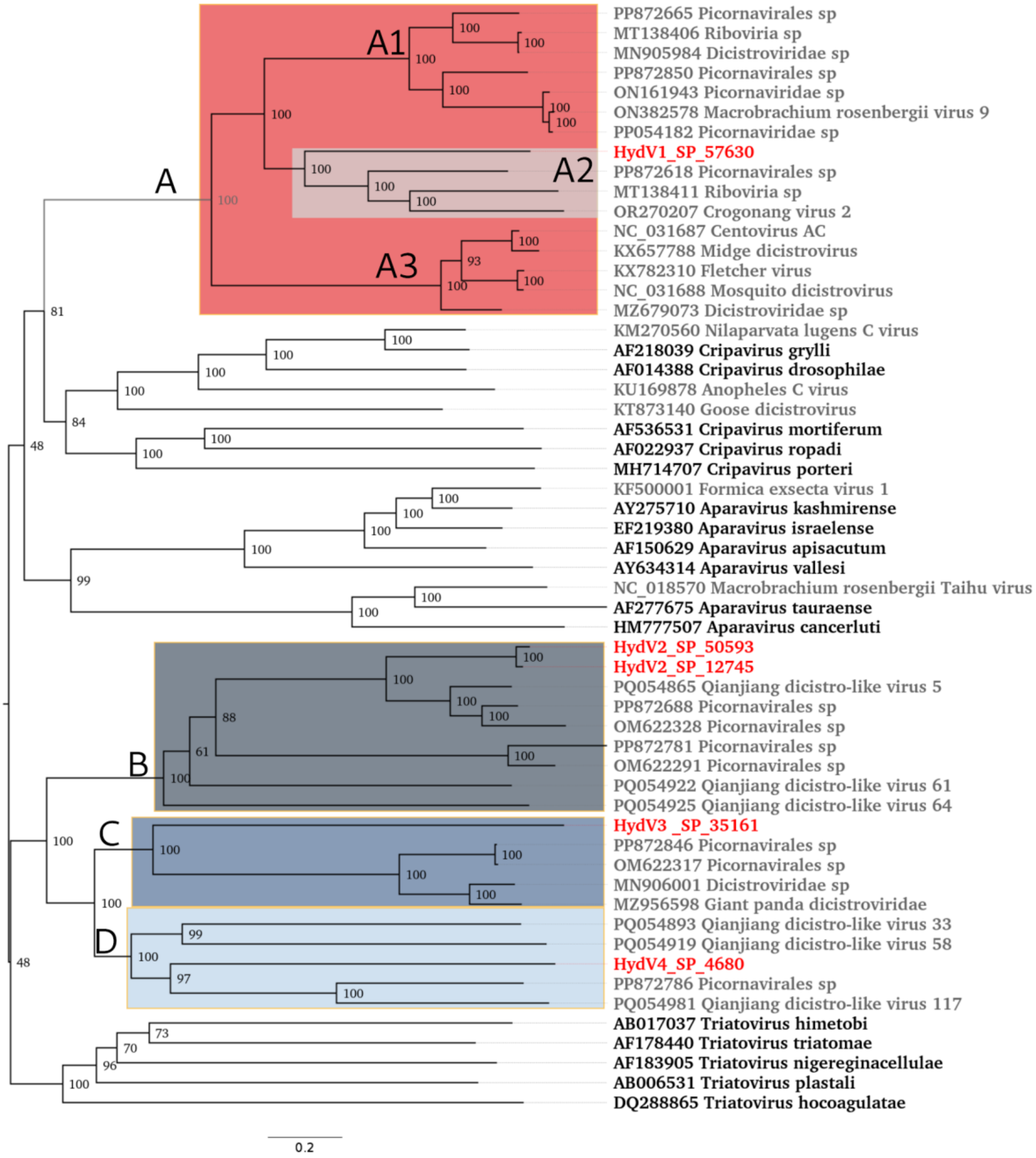


**Figure S4.** Maximum likelihood trees of the complete genomes . HydV are in red font, classified viruses in black, unclassified viruses in gray. The three clades into which the sequences clustered are highlighted by a square. The number at each node indicates the bootstrap probability (%).

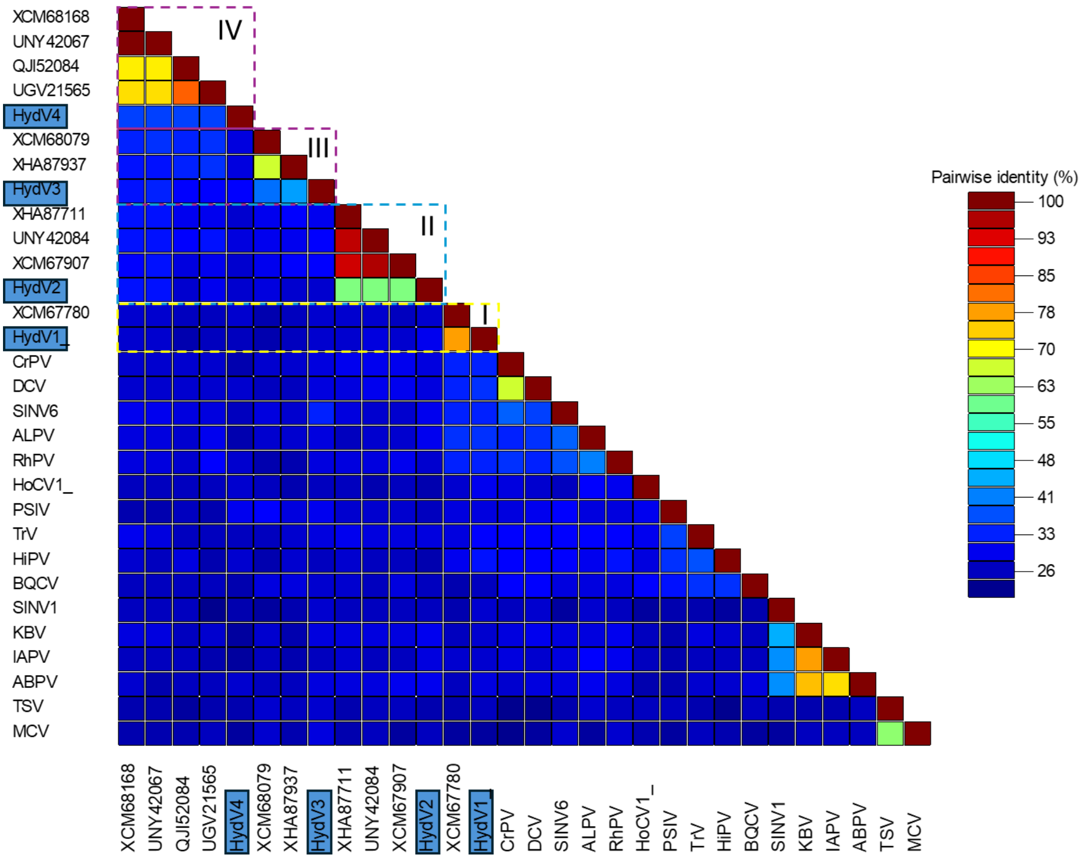


**Figure S5**. Pairwise identity matrix of HydV sequences (ORF2).

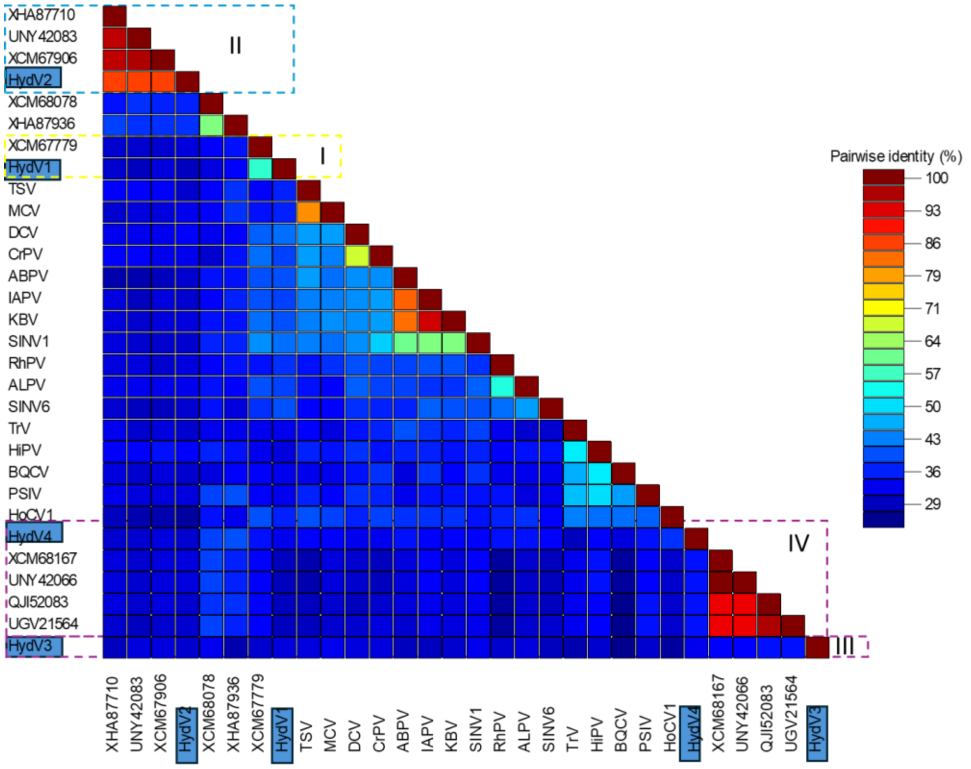


**Figure S6**. Pairwise identity matrix of HydV sequences (ORF1).
